# Applications of Gold Nanoparticles in Non-Optical Biosensors

**DOI:** 10.3390/nano8120977

**Published:** 2018-11-26

**Authors:** Pengfei Jiang, Yulin Wang, Lan Zhao, Chenyang Ji, Dongchu Chen, Libo Nie

**Affiliations:** 1 Hunan Key Laboratory of Biomedical Nanomaterials and Devices, Hunan University of Technology, Zhuzhou 412007, China; m16077700001@stu.hut.edu.cn (P.J.); m18077700009@stu.hut.edu.cn (Y.W.); m18077700015@stu.hut.edu.cn (L.Z.); 16420200115@stu.hut.edu.cn (C.J.); 2 School of Material Science and Energy Engineering, Foshan University, Foshan 528000, China; chendc@fosu.edu.cn

**Keywords:** gold nanoparticles, biosensor, piezoelectric, electrochemical, ICP-MS

## Abstract

Due to their unique properties, such as good biocompatibility, excellent conductivity, effective catalysis, high density, and high surface-to-volume ratio, gold nanoparticles (AuNPs) are widely used in the field of bioassay. Mainly, AuNPs used in optical biosensors have been described in some reviews. In this review, we highlight recent advances in AuNP-based non-optical bioassays, including piezoelectric biosensor, electrochemical biosensor, and inductively coupled plasma mass spectrometry (ICP-MS) bio-detection. Some representative examples are presented to illustrate the effect of AuNPs in non-optical bioassay and the mechanisms of AuNPs in improving detection performances are described. Finally, the review summarizes the future prospects of AuNPs in non-optical biosensors.

## 1. Introduction

Nanoparticles are defined as particles with sizes between 1 and 100 nm. Due to their physical and chemical properties such as high specific surface area, electrical performance, magnetism, optical and catalytic property, nanoparticles have received great attention in many research fields [1,2]. Especially, AuNPs have excellent properties such as good biocompatibility, excellent conductivity, effective catalysis, high density, and high surface-to-volume ratio, which are widely used in the field of bioassay [3,4,5,6,7,8,9].

As one of the most stable metal nanoparticles, AuNPs play an important role in the field of biosensors. AuNPs can be easily modified with biomolecules such as DNAs and proteins by thiol and amine via Au-S or Au-N bonds without destroying the activity of biomolecules. In optical biosensors, AuNPs are widely used to improve the detection sensitivity of fluorescence, chemiluminescence, surface plasmon resonance (SPR), surface-enhanced Raman scattering (SERS) method, and other optical detection [10,11,12,13]. The AuNPs are usually used as fluorescence quenchers, catalysts, immobilization platforms, colorimetric nanoparticles, as well as SPR and SERS enhancers in optical biosensors. It shows that the sensitivities of optical biosensors are effectively improved based on the signal amplification of AuNPs. However, the optical detections usually require expensive instruments such as fluorescent spectrometers, SPR/SERS instruments, which increases the cost of bioassay.

In non-optical biosensors, AuNPs are mainly used in piezoelectric biosensors, electrochemical biosensors and ICP-MS biosensors. In piezoelectric biosensors, AuNPs usually act as labels which make use of their high density to increase the mass change and improve the sensitivity of detection [14,15]. In electrochemical biosensors, AuNPs are often used as immobilization platform, electrocatalyst or electron migration enhancer which exhibit advantages in improving the sensitivity, selectivity and stability of detection [16,17,18]. In recent years, AuNPs have also been reported in biological detection based on ICP-MS technology [19,20,21,22]. Similar to optical biosensors, the performances of non-optical biosensors are effectively improved based on the signal amplification of AuNPs. Although the instruments of non-optical detection are simple, the detection procedures are not as automatic and rapid as those of the optical biosensors, which are not widely used in clinic application.

In this review, we focus on the applications of AuNPs in non-optical bioassay strategies of piezoelectric biosensors, electrochemical biosensors and ICP-MS detections. The effects of AuNPs in these detection methods are described. Finally, we summarize the future prospects of AuNPs in non-optical biosensors.

## 2. Piezoelectric Biosensors

The most common type of piezoelectric biosensor is quartz crystal microbalance (QCM), which is a sensitive technique based on the piezoelectric effect [23]. When a mechanical force is exerted on the quartz crystal, the crystal generates an electric potential in the direction of the applied force. Oppositely, when an electric field is applied to the crystal, the crystal generates mechanical vibrations. When a certain substance is adsorbed on the surface of quartz crystal, the resonance frequency of the crystal will shift from its basic frequency. Therefore, the mass change on the surface of quartz crystal can be detected by the frequency shift according to piezoelectric effect. QCM biosensors possess the advantages of high sensitivity at a nanogram level, label-free and real-time monitoring, which is widely used in the detection of genes, proteins, cells, microorganisms, toxins and so on [24,25,26,27,28,29,30,31,32,33,34,35,36,37,38,39,40,41,42,43,44].

Because of their large specific surface area, AuNPs are often immobilized on the surface of the quartz crystal in order to connect more biomolecules in QCM biosensors. Jiang’s team reported that AuNPs were immobilized on the surface of a gold electrode to increase the number of capture probes and hybridize more target DNAs. It suggested that the sensitivity of this method is three times more than that without AuNP immobilization [45]. Moreover, they deposited AuNPs on the surface of platinum coated QCM (Pt-QCM) to provide more binding sites for HS-DNA, and the maximum immobilization amount of HS-DNA on (Au)Pt-QCM was about three-fold more than that on bare Pt-QCM [46]. In addition, to immobilize more AuNPs on the surface of the crystal, they developed a novel method that a large amount of AuNPs were adsorbed on the surface of polystyrene microspheres which were immobilized on the surface of Au electrode, with a low detection limit of 10^−12^ M for DNA analysis [47].

**Figure 1:**
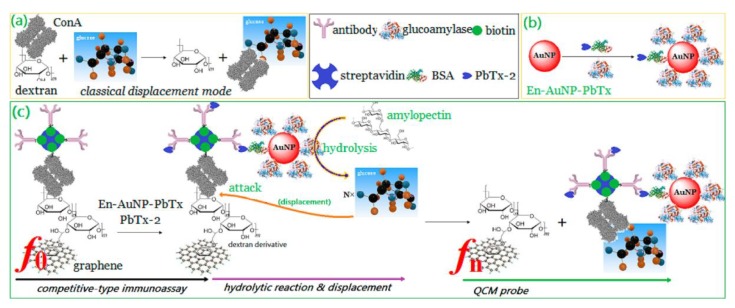
(**a**) Conventional displacement-type assay protocol based on the dextran-concanavalin A (ConA)-glucose system, (**b**) schematic illustration of gold nanoparticle heavily functionalized with glucoamylase and PbTx-2 BSA, and (**c**) measurement principle of the displacement-type QCM immunosensor. Reproduced with permission from [57]. American Chemical Society, 2013.

Obviously, AuNPs have a significant signal amplification effect in QCM biosensors due to their heavier masses than biomolecules. Owing to their high density, AuNPs have potential as labels to increase the mass change on the quartz surface [48,49,50]. Jiang et al. used AuNPs of 50 nm as the mass enhancer to amplify the frequency signal of QCM, which reached a low detection limit of 10^−14^ M for target DNA [51]. Furthermore, they improved the detection limit to 10^−16^ M by modifying AuNPs on the surface of gold electrode and labeling AuNPs with probe DNA simultaneously [52,53]. Premaratne et al. carried out a similar research and obtained an ultralow detection limit of 28 fM for target oligonucleotide [54]. To further increase the frequency shift, Chen et al. exploited a QCM-DNA sensor with a layer-by-layer AuNPs structure by DNA hybridization, which achieved an ultralow detection limit of 2 plaque forming units (PFU)/mL for dengue virus (DENV) [55]. Kim et al. found that the introduction of AuNP modified antibodies increased the signal by 53.4% compared with that without AuNPs modification [56]. Tang and co-workers proposed a novel displacement-type QCM immunosensor based on AuNPs, which lead to a significant frequency shift, and a detection limit as low as 0.6 pg·mL^−1^ for brevetoxin B (PbTx-2) (Figure 1) [57].

The technique of gold label silver stain (GLSS) is also an excellent choice to enhance the mass change in QCM biosensor. In the presence of reducing agents such as hydroquinone quinol (HQ), AuNPs catalyze the reduction of silver ions to form silver element which deposits on the surface of AuNPs to obtain Au@Ag core-shell structure, which highly increases the mass change and improve the detection sensitivity. Shan et al. constructed a QCM cell sensor based on the classical GLSS signal amplification method, and the limit of detection (LOD) for acute leukemia cells was 1160 cells·mL^−1^ [58].

Another effective way to improve the sensitivity of QCM biosensors is to combine AuNPs with biological amplifying technologies. Sun et al. developed a method with multi-cycle signal amplification based on AuNPs and hybridized chain reaction (HCR), which the large number of AuNPs were assembled on the HCR products for signal amplification, and the detection limit of the target DNA was as low as 0.7 fM [59].

## 3. Electrochemical Biosensors

Electrochemical biosensors show the advantages of high sensitivity, low-cost, amenable miniaturization and operating convenience. AuNPs play an important role in improving the sensitivity and specificity of electrochemical biosensors, such as modifying the sensing surface to enhance conductivity, increasing the immobilization of biomolecules and catalyzing the electrochemical reactions. In addition, AuNPs are also used as the electrochemical indicators.

### 3.1. AuNPs as the Electrochemical Indicators

AuNPs can be used as electrochemical indicators based on the redox reaction between Au^0^ and Au^3+^ [60]. In electrochemical biosensors, the ways to detect AuNPs signal mainly include: (i) direct detection of the oxidation signal of AuNPs without treatment [61]. (ii) AuNPs are electro-oxidized to gold ions in hydrochloric acid (HCl) solution [62,63,64,65,66,67]. (iii) AuNPs are dissolved in HBr/Br_2_ acidic solution [68,69].

**Figure 2:**
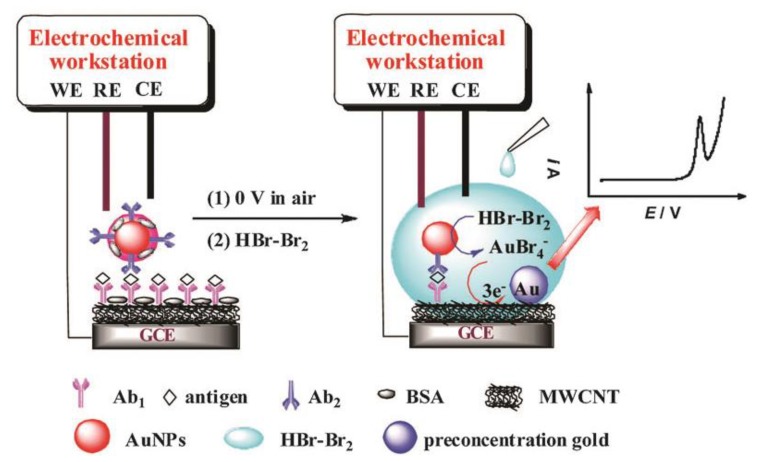
Illustration of key electrochemical steps of the metal-labeled amperometric immunoassay signal amplification protocol. Reproduced with permission from [73]. Royal Society of Chemistry, 2015.

Kerman et al. reported an electrochemical sensor for DNA detection by the direct oxidation of AuNPs without acid treatment, which provided a detection limit of 2.17 pM for target DNA [70]. Although it is simple to detect AuNPs directly, most of AuNPs cannot be detected due to their distances away from electrodes. Therefore, AuNPs are usually oxidized to gold ions in most electrochemical detections. Trau et al. reported a fast and sensitive electrochemical detection in which the AuNPs were electrochemically oxidized to Au^3+^ in HCl solution at first, and then the reduction of gold ions was detected which obtained a detection limit as low as 1 colony-forming units (CFU) for *Mycobacterium tuberculosis* (*Mtb*) DNA [71]. Ilkhani et al. designed an electrochemical sandwich immunosensor in which cathodic preconcentration and anode stripping of gold were performed after AuNPs were dissolved in HCl solution. The detection limit of this method was 50 pg·mL^−1^ [72]. Preconcentration of gold ions on the surface of electrodes can increase the recovery rate of AuNPs and enhance the electrochemical signals. Qin et al. reported a new method by cathodic preconcentration of gold ions. Unlike traditional AuNP electrochemical measurements, a cathodic potential (0 V here) was firstly applied on the electrode in air, and then the dissolution of AuNPs and cathode preconcentration simultaneously performed in microliter-droplet aqueous HBr/Br_2_. This scheme presented high signal recovery efficiency of AuNPs, and the detection limit was as low as 0.3 fg·mL^−1^ for human immunoglobulin G (hIgG) and 0.1 fg·mL^−1^ for the human prostate-specific antigen (hPSA) (Figure 2) [73].

**Figure 3:**
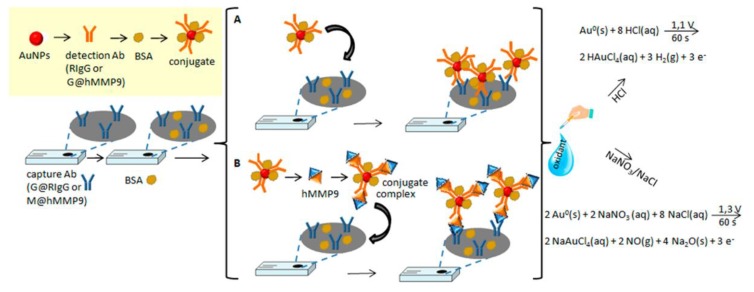
Scheme of the preparation of the two immunosensors used and their analytical working principle. (**A**) model system and (**B**) immunosensor for hMMP9 detection. Reproduced with permission from [74]. American Chemical Society, 2018.

The corrosive solutions such as HBr/Br_2_ and HCl used in dissolving AuNPs are harmful to the ecological environment and human health. Therefore, green reagents are needed to replace acidic electrolytes for AuNP electrooxidation. Recently, NaNO_3_/NaCl mixture was first proposed by Baldrich et al. as a potential alternative. The results showed that NaNO_3_/NaCl mixture exhibited better electrooxidation performance than other oxidized salts, but the reduction peak is much lower than that of the HCl solution. It’s also suggested that NaNO_3_/NaCl provided nanoimmunoconjugate quantization in all the concentration range. Therefore, NaNO_3_/NaCl can be used to substitute HCl, providing a more environmentally friendly method for electrochemical measurement of AuNPs (Figure 3) [74].

### 3.2. AuNPs as the Electron Migration Enhancers

In electrochemical biosensors, the electrochemical redox reaction generates electron exchange on the electrode, which is relative to the concentration of the analytes. However, the direct electrochemical detection is often difficult to achieve because of the weak electrical conductivity of biomolecules which blocks the transfer of electrons to the electrode. In order to enhance the conductivity, AuNPs are usually immobilized on the surface of the electrode, which not only enhances the electron transfer rate in the electrochemical process, but also enlarges the sensing area to increase the immobilized amount of the recognition unit, thereby achieving the improvement of sensitivity.

**Figure 4:**
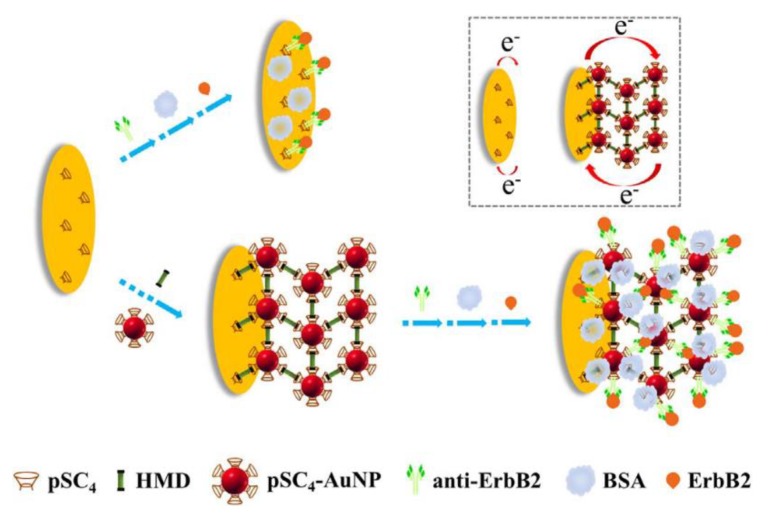
Schematic of pSC_4_ monolayer and pSC_4_-gold nanoparticles (AuNPs) layer-by-layer signal amplification on the electrode surface. Reproduced with permission from [84]. Elsevier, 2018.

As early as 1996, Natan’s group had demonstrated the direct electron transfer of AuNPs between proteins and electrodes [75]. Since that, much research using AuNPs as the electron migration enhancer has been published [76,77,78,79,80]. Electrodeposition is a common method to immobilize AuNPs on the surface of electrodes. Zhao et al. electrochemically deposited AuNPs on the surface of glassy carbon electrode (GCE), and a wide linear range from 0.5 pg·mL^−1^ to 100 ng·mL^−1^ and the ultralow detection limit of 145.69 fg·mL^−1^ was achieved for prostate-specific antigen (PSA) detection [81]. Bao et al. also used AuNPs deposition modified GCE to detect DNA methylation and DNA methyltransferase [82]. Another way to modify AuNPs on the surface of electrodes is the direct immobilization of AuNPs. Jarocka et al. immobilized AuNPs on the surface of gold electrode as the electron migration enhancer, achieving a LOD of 2.2 pg·mL^−1^ for target protein [83]. The size of AuNPs has influence on the performance of the biosensor, affecting mainly linearity of the output signal and reproducibility of assays. To immobilize more AuNPs on the electrode surface, a three-dimensional structure of AuNPs was developed by Wang’s group. They designed a layer-by-layer assembly of AuNPs on the surface of electrode by *para*-Sulfonatocalix[4]arene (pSC_4_) modified AuNPs and 1,6-hexanediamine (HMD) conjugation through host-guest recognition. With enhanced electron migration and large specific surface area of AuNPs, this structure showed a detection limit of 0.5 ng·mL^−1^ for human epidermal growth factor receptor 2 (ErbB_2_) (Figure 4) [84].

To enhance the conductivity furthermore, AuNPs are also combined with other high conductive materials such as graphene, carbon nanotubes and dendrimers in electrochemical biosensor. Wang et al. developed an electrochemical DNA sensor in which the chitosan-graphene sheet and polyaniline were modified on the surface of GCE to increase the effective surface area of the electrode to deposit more AuNPs. The detection limit of this method was as low as 2.11 pM [85]. Gao et al. reported an electrochemical immunosensor based on AuNPs and Nile blue A (NB) hybridized electrochemically reduced graphene oxide (NB-ERGO). In this study, NB-graphene oxide (NB-GO) and HAuCl_4_ were simultaneously reduced to synthesize AuNPs/NB-ERGO on the surface of the electrode, which provided a large surface area for antibody attachment, achieving a detection limit of 1 pg·mL^−1^ for carcinoembryonic antigen (CEA) [86]. Furthermore, to enhance the specific surface area of electrode, Shuai et al. proposed an ultrasensitive electrochemical biosensor by combining tungsten oxide-graphene (WO_3_-Gr) composites with AuNPs on the electrode to provide more binding sites for the probes, obtaining a detection limit of 0.05 fM for microRNA [87]. Nanocarbon materials are often used in electrochemical biosensors due to their good conductivity. Bai et al. attached the electrode by single-walled carbon nanotube modified AuNPs to improve the conductivity of the electrode and provide more binding sites for biomolecules, receiving a detection limit of 8 pM for platelet-derived growth factor (PDGF) and 11 pM for thrombin respectively [88]. Liu et al. produced a composite of AuNPs coated with graphitized mesoporous carbon nanoparticles for the detection of PSA. This composite increased the electron transfer rate and the immobilizing number of aptamers on the surface of electrode, resulting in a limit of detection less than 0.25 ng·mL^−1^ and a linear detection range from 0.25 to 200 ng·mL^−1^ [89]. In addition, dendrimer-encapsulated AuNPs are also developed to enhance the signal of electrochemical biosensors, which possess the advantages of high density of active groups, excellent structural homogeneity, good biocompatibility and conductivity. Jeong et al. reported the poly(amidoamine) dendrimer encapsulated AuNPs (PAMAM-AuNPs) for CEA detection, which not only increased the immobilized amount of the antibody, but also accelerated the electron transfer process, resulting in a linear dynamic range of 10.0 pg·mL^−1^ to 50.0 ng·mL^−1^ and a detection limit of 4.4 pg·mL^−1^ [90]. Zhang et al. also exploited a highly sensitive electrochemical immunosensor based on PAMAM-AuNPs with a detection limit of 50 CFU·mL^−1^ for *Escherichia coli* (*E. coli*) [91].

**Figure 5:**
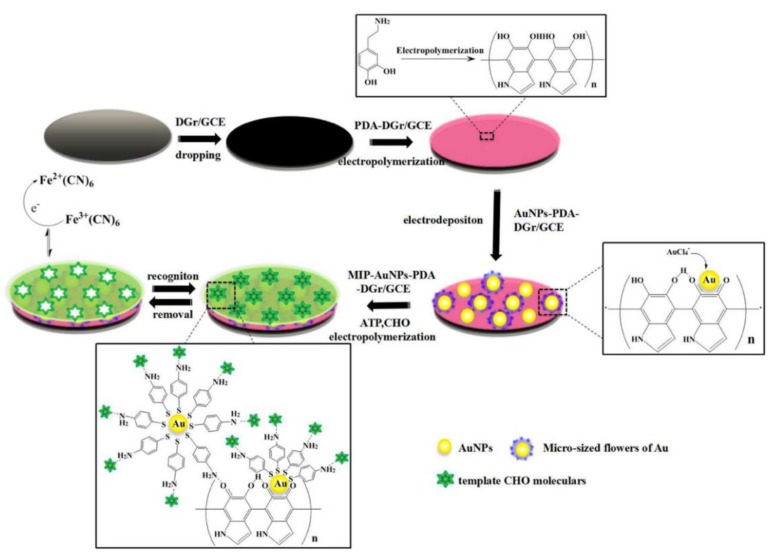
The preparation process of MIP-AuNPs-PDA-DGr/GCE. Reproduced with permission from [93]. Elsevier, 2017.

In addition, AuNPs are also used in molecularly imprinted electrochemical biosensors by increasing the surface area of the recognition unit and improving the conductivity of the molecularly imprinted polymer (MIP) film [92]. Yang and co-workers developed a novel molecularly imprinted electrochemical sensor for cholesterol (CHO) detection based on bioinspired Au microflowers. In this study, the bioinspired Au microflowers were formed on the surface of the electrode by wrapping AuNPs on the bioinspired polydopamine (PDA) film through electropolymerization, followed by the coating of MIP. The linear response range of this strategy was between 10^−18^ and 10^−13^ M, with an ultralow detection limit of 3.3 × 10^−19^ M, which is more sensitive than the traditional CHO detection method (Figure 5) [93].

The regeneration of biosensor is able to simplify operation, reduce cost and save time, which is favourite in the detection process. Sun et al. developed an ultrasensitive electrochemical biosensor for the detection of human liver hepatocellular carcinoma (HepG2) cells. After measurement, an electrochemical reductive desorption method was performed to break gold thiol bond and desorb the components on the surface of AuNPs/GCE, which retained 90% of the original sensitivity [94].

### 3.3. AuNPs as the Immobilization Platform

For electrochemical biosensors, the number of electroactive molecules is a key factor to the detection sensitivity, which is usually improved by increasing the amount of electrochemical signal molecules through various amplifying strategies. Because of the advantages of large specific surface area and easy conjugation with biomolecules by Au-S bond, AuNPs are usually used as the immobilization platform to connect a large number of biomolecules, resulting in the conjugation of a great deal of electrochemical signal molecules [95,96,97,98,99,100,101,102,103,104].

Wang and co-workers developed an electrochemical DNA sensor based on the amplification of AuNPs. In this method, a large number of the methylene blue (MB) labeled DNA probes were immobilized on the surface of AuNPs. Attribute to the large specific surface area of AuNPs, the electrochemical signals of MB were effectively amplified, resulting in a detection range of 10^−13^ to 10^−8^ M and a detection limit as low as 50 fM [105]. Shu et al. modified 6-ferrocenyl hexanethiol (Fc) and aptamers on the surface of AuNPs simultaneously that the amount of the former was much more than that of the latter. After the biorecognition of aptamers and CEA, the amplified electrechemical signal of Fc significantly improved the detection sensitivity [106]. Hasanzadeh et al. used AuNPs to support histidine (nano-Au-Hist), which showed a perfect discriminatory power for the Brucella-specific probe hybridization [107]. Wang et al. reported AuNPs as the platform to immobilize DNAs for the detection of ampicillin [108]. In addition, AuNPs often combined with other nanomaterials to further improve the performance of the biosensors. Chen et al. reported that the AuNPs were grown on the surface of the octahedral Cu_2_O nanocrystals to increase the surface area and immobilize recognition components and electroactive substances, which presented a detection limit as low as 23 fM for thrombin (TB) [109].

**Figure 6:**
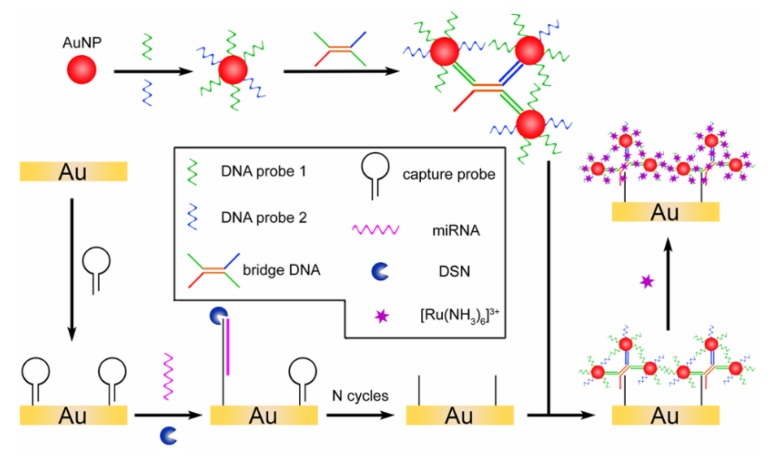
Illustration of the electrochemical approach for triple amplified detection of miRNA. Reproduced with permission from [111]. American Chemical Society, 2018.

Combining AuNPs with signal amplifying technologies is a common way to increase the detection sensitivity of electrochemical biosensors. Zhu’s group designed an electrochemical detection strategy based on spherical nucleic acids AuNPs triggered mimic-hybridization chain reaction (mimic-HCR). The AuNP carried DNA probes initiated the mimic-HCR which the double-stranded structure bound a large amount of [Ru(NH_3_)_6_]^3+^ to amplify the electrochemical signal [110]. Recently, Bo et al. developed a triple-signal amplification method for the determination of miRNA. In this protocol, AuNPs were connected together to form the bridge DNA-AuNPs nanocomposites, which was used to absorb a large number of electrochemical indicator [Ru(NH_3_)_6_]^3+^. This strategy achieved a wide detection linear range of 10^−17^ to 10^−11^ M, with limit of detection as low as 6.8 aM (Figure 6) [111]. Yu et al. developed AuNPs hot-spots self-assemble structure by catalytic hairpin assembly (CHA) reaction to improve the absorption amount of [Ru(NH_3_)_6_]^3+^, obtaining a detection limit as low as 25.1 aM for miRNA-141 [112]. It suggests that AuNPs combined with bio-amplification technologies such as bio-barcode HCR, CHA lead to a detection limit as low as aM level, which is of great prospect to enhance the sensitivity of electrochemical biosensors.

**Figure 7:**
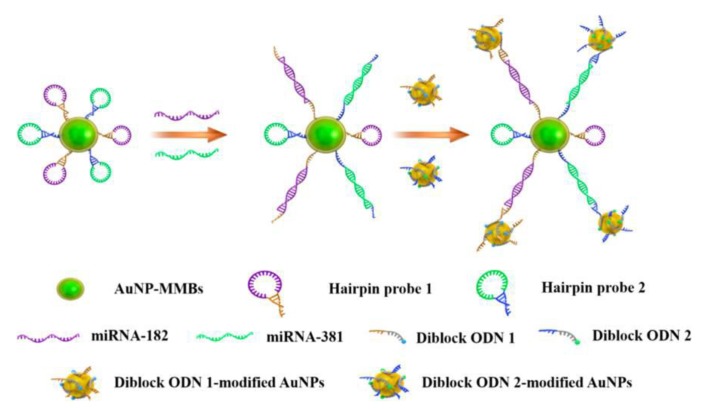
Schematic showing the simultaneous electrochemical detection of miRNA-182 and miRNA-381 via the conjugates of AuNP-MMBs and diblock ODN-modified AuNPs. Reproduced with permission from [113]. American Chemical Society, 2017.

In addition, Wang et al. reported a multiple electrochemical detection method for quantitative analysis of miRNAs. In this work, gold nanoparticle-coated magnetic microbeads (AuNP-MMBs) were used as the carrier to connect two hairpin probes. At the same time, the electrochemical indicators MB and Fc modified diblock oligonucleotides (ODNs) were immobilized on the surface of AuNPs as the signal output. Two target miRNAs were detected simultaneously with detection limits as low as 0.2 fM and 0.12 fM for miRNA-182 and miRNA-381 respectively (Figure 7) [113].

### 3.4. AuNPs as the Catalyst

Bulk gold is chemically inert, while AuNPs exhibit extraordinary catalytic capability [114,115,116,117]. Studies show that the catalytic activity of AuNPs arises from their quantum scale, high surface-to-volume ratio and interface-dominated property, which is able to reduce the overpotential of electrochemical reactions and accelerate the chemical reaction, leading to the improvement of detection sensitivity. Typically, AuNPs are used to catalyze redox reactions such as nicotinamide adenine dinucleotide (NADH), hydrogen peroxide (H_2_O_2_), 4-nitrophenol, o-phenylenediamine (o-PD), catechol and nitrite [118,119,120].

Raj et al. described the electrocatalytic oxidation effect of AuNPs on NADH. The self-assembly of AuNPs on a thiol-terminated three-dimensional silicate network modified on the surface of the electrode catalyzed the oxidation of NADH, reducing the overpotential by 915 mV without any electron transfer mediator [121]. Li et al. prepared a sandwich immunosensor for the analysis of alpha fetoprotein (AFP). In this study, AuNPs functionalized magnetic multi-walled carbon nanotubes (MWCNTs-Fe_3_O_4_) were utilized to adsorb lead ions and antibodies, which exhibited good electrocatalytic activity for the reduction of H_2_O_2_. Under optimal experimental conditions, the detection limit reached 3.33 fg·mL^−1^ for AFP [122]. Cao et al. structured an AuNP network to catalyze the redox of H_2_O_2_ and HQ, with a detection limit of 0.32 pM for lysozyme [123].

To improve the electrochemical signals, considerable research efforts have been devoted to the application of GLSS in electrochemical biosensors [124,125,126,127,128,129,130,131]. Because of the amplification of GLSS, the electrochemical signal of silver is highly improved, which leads to the enhancement of detection sensitivity. Lai et al. constructed an electrochemical immunoassay strategy based on GLSS for the detection of human and mouse IgG [132]. The amount of AuNPs is one of the keys to enhance the effect of GLSS. Recently, Zhang et al. used polypyrrole microsphere (PPyMS) to immobilize more AuNPs for the amplification of silver label signal. A low detection limit of 0.1 ng·L^−1^ and a wide linear range of 0.25 ng·L^−1^ to 50·L^−1^ was obtained for microcystin-LR (MC-LR) detection [133].

**Figure 8:**
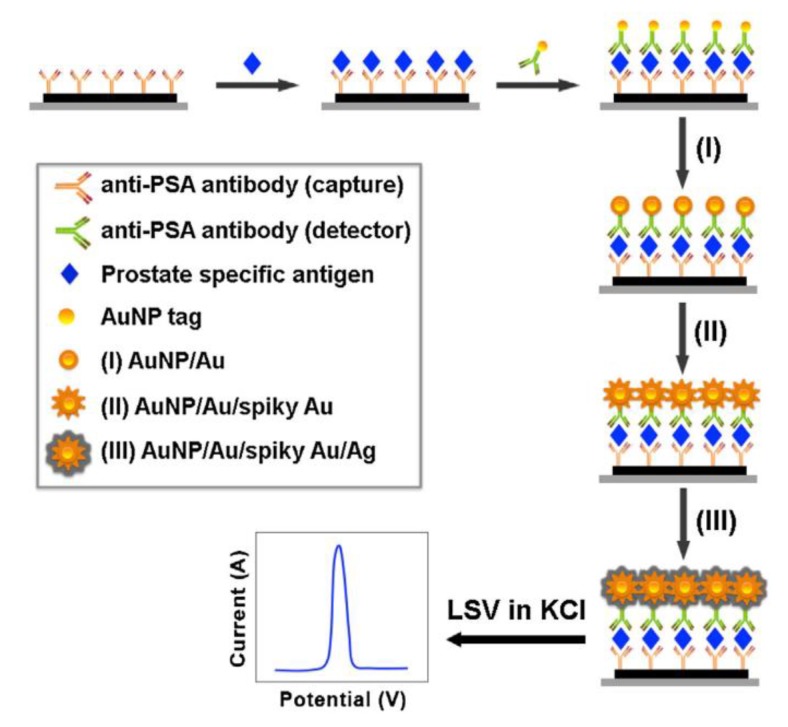
Schematic representation of triple signal amplification strategy based on AuNPs serving as labeling tags. Sandwich immunoreaction of PSA was used as an immunosensing model. Linear sweep voltametric analysis was performed to detect deposited silver. Reproduced with permission from [135]. Elsevier, 2017.

Combining AuNPs with other nanomaterials is also an effective way to improve the performance of electrochemical biosensors. Yang et al. used AuNPs functionalized nitrogen-doped graphene quantum dots (Au@N-GQDs) to enhance conductivity and synthesized the echinoidea-shaped nanocomposites (Au@Ag-Cu_2_O) which composed of Au@Ag core-shell nanoparticles and disordered cuprous oxide to label antibodies. Taking advantages of the conductivity and catalysis of AuNPs, an ultralow detection limit of 0.003 pg·mL^−1^ for PSA was achieved [134]. Studies show that the size of AuNPs affects the catalysis of silver deposition, and relatively high deposition currents of silver can be obtained using small AuNPs. Duangkaew et al. developed a triple signal amplification strategy based on small-sized gold nanoparticles for the electrochemical detection of PSA. In this method, the size of AuNPs was increased by forming an Au shell on the surface of the small AuNP tags, and then the spiky AuNPs were grown on the surface of Au shell, with the benefit of enhancing the catalysis of silver. Compared to the traditional GLSS process, this triple signal amplification strategy magnified the electrical signal by 260 times (Figure 8) [135].

The electrochemical biosensors based on AuNPs are summarized in Table 1.

## 4. ICP-MS Biosensor

Inductively coupled plasma mass spectrometry (ICP-MS) combines the high-temperature ionization characteristics of inductively coupled plasma with the sensitive and fast scanning of mass spectrometers, which is a high sensitive technique for element, isotope and morphological analysis [136]. The technology offers extremely low detection limit and an extremely wide dynamic linear range with a working range more than 9 orders of magnitude, and owns the advantages of simple spectral lines, low interference, high analytical precision, rapid analysis and high specificity, which is widely used in environmental protection, biology, medicine, metallurgy, nuclear material analysis and other fields [137,138,139,140,141,142,143]. In the past few years, the strategies combining ICP-MS technology with metal nanoparticle labels were developed to achieve ultra-high sensitivity analysis in biomolecular analysis [144,145,146,147,148,149,150,151,152].

**Figure 9:**
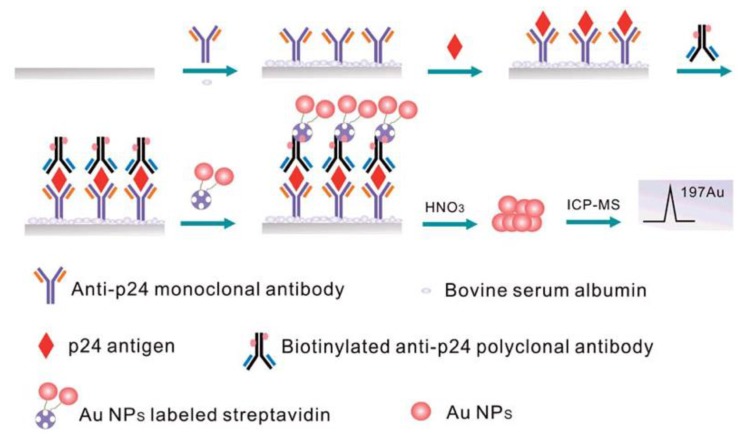
Schematic diagram of the sensitive assay with the BA system and Au NPs based immunoassay for p24 antigen determination by ICP-MS. Reproduced with permission from [153]. Royal Society of Chemistry, 2014.

AuNPs are composed of plenty of gold atoms, which generate a huge number of Au ions by dissolution, digestion and plasma. Using AuNPs as the labels, the ultra-high sensitive detection of biomolecules is able to achieve by ICP-MS technology. He et al. developed an ICP-MS biosensor based on AuNP labels for HIV-1 p24 antigen detection. In this study, diluted HNO_3_ was used to dissociate AuNPs from the immunoassay complex, with a detection limit of 1.49 pg·mL^−1^ by ICP-MS measurement (Figure 9) [153]. Similar methods were developed in cell and immune assay [154].

**Figure 10:**
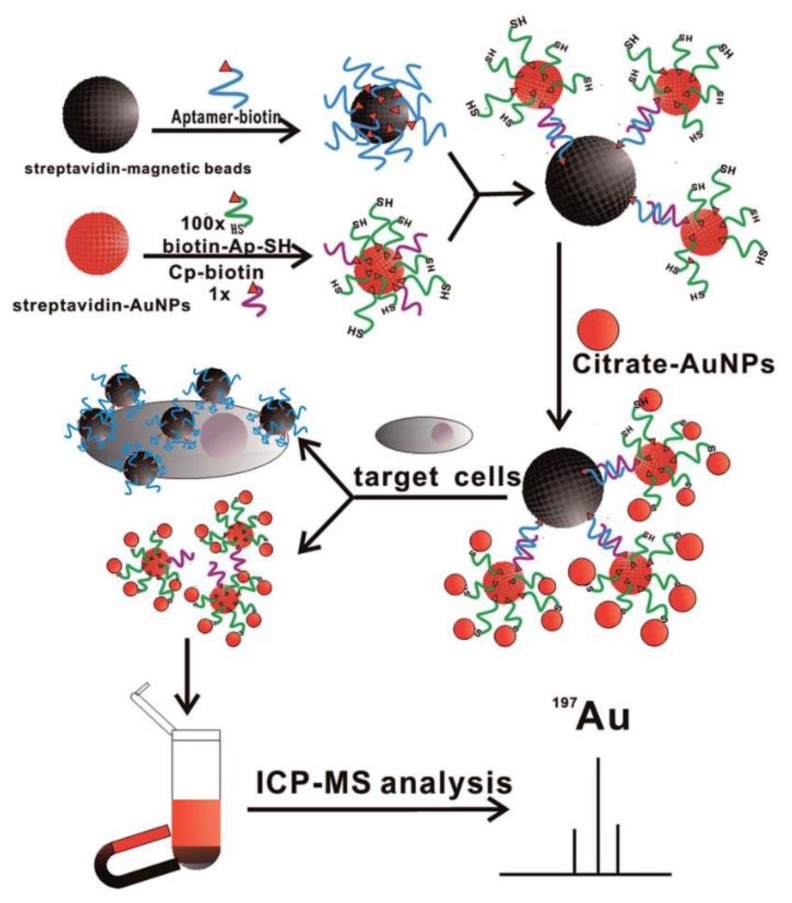
Schematic illustration of the experimental principle for counting cancer cells based on ICP-MS detection and a MB-based AuNP aptamer labelling technique. Reproduced with permission from [155]. Royal Society of Chemistry, 2016.

To improve the signal of ICP-MS measurement, the amplification methods have been developed to increase the amount of labeled AuNPs. Yang et al. reported a layer-by-layer assembly method of AuNPs to amplify the ICP-MS signals for the detection of cancer cells. The detection limit of human hepatocellular carcinoma SMMC-7721 cells was as low as 100 cells·mL^−1^ (Figure 10) [155]. Li et al. developed an ICP-MS ultrasensitive immunoassay based on AuNPs and tyramine signal amplification (TSA), with a detection limit of 1.85 pg·mL^−1^ for AFP [156]. He et al. reported a new method based on rolling circle amplification (RCA) and ICP-MS detection, which provided an ultralow detection limit of 0.1 fM and a good specificity [157]. Zhang et al. reported an AuNPs labelling and HCR amplification strategy for HepG2 cells detection by ICP-MS, with detection limit as low as 15 cells and a linear range of 40–8000 cells [158]. Li et al. reported a triple signal amplification strategy based on AuNPs, which combined RCA, nicking displacement and bio-bar-code techniques to perform ultra-sensitive detection of target DNA by ICP-MS. This strategy provided a detection limit as low as 3.2 × 10^−17^ M for hepatitis B virus (HBV) DNA [159]. Liu et al. reported a novel strategy based on capillary electrophoresis and inductively coupled plasma mass spectrometry (CE-ICP-MS). The results shown that more than 2000 Au atoms were attached to each albumin with a detection limit as low as 0.1 aM and a wide linear range of 4 orders of magnitude [160].

Conventional ICP-MS is usually used in analyzing the concentrations and compositions of the elements. However, single-particle mode ICP-MS (sp-ICP-MS) is able to perform multiple information analysis on the structure, shape, particle size, and agglomeration of nanoparticles, which extends the application of ICP-MS technology in bioassay [161,162,163,164,165,166,167,168,169,170]. For the first time, Allabashi et al. evaluated the possibility of direct determination of AuNPs in colloid solutions by ICP-MS, without previous digestion/dissolution. The results showed no significant difference compared to the same AuNPs by acidic digestion [171]. Liu et al. reported that ICP-MS was able to measure AuNPs with the sizes from 10 to 70 nm under high sensitive mode, and the size of AuNPs could be further extended to 200 nm in less sensitive mode [172]. In sp-ICP-MS detection, the frequency of the pulse signal is a function of the concentration of AuNP colloids and the recorded peak distribution of signal intensity is a function of size distribution. It can be used in the detection of biomolecules. Han et al. reported a DNA detection method based on AuNPs and sp-ICP-MS. In this method, the hybridization of DNA targets with DNA probes immobilized on the surface of the AuNPs resulted in the formation of dimers, trimers, or even large aggregates of AuNPs. This polymeric network aggregation led to decreased concentrations of the whole AuNP population as well as increased individual sizes. These changes were detected by sp-ICP-MS quantitatively, and thus the amount of DNA was obtained. The quantitative detection of AuNPs aggregates was performed directly and yielded a good linear relationship, with a LOD as low as 1 pM [173]. Therefore, the sp-ICP-MS is a powerful tool for nanoparticle detection, which is environmentally friendly, and needn’t use toxic reagents such as HCl and nitric acid to digest AuNPs.

## 5. Conclusions

Nanotechnology promotes the development of many fields such as bioassay and biorecognition [174,175,176,177,178,179,180,181,182,183,184,185,186,187]. Nanomaterials play a crucial role in enhancing the performance of biosensors. In non-optical bioassay, AuNPs are widely used to improve the detection sensitivity due to their good physical and chemical properties. Taking advantage of their heavy mass, AuNPs are utilized to increase the mass change and improve the frequency shift in piezoelectric biosensor. In electrochemical biosensor, AuNPs are used as an electrochemical indicator, electron migration enhancer and catalyst based on their excellent conductivity and effective catalysis. In addition, the gold element in AuNPs can be detected by ICP-MS technology. Because of their high specific surface area, AuNPs are often used as the immobilization platform to immobilize more biomolecules and enhance the detection sensitivity. Combining AuNPs with other various signal amplifying methods such as RCA, HCR, bio-barcode and layer-by-layer assembly, the sensitivities of biosensors are highly improved to achieve detection limits as low as attomole or below. Although AuNPs perform excellently in improving the sensitivity of non-optical biosensors, there are still challenges to be faced. The potential of AuNPs in non-optical bioassay should be further explored to design new bioassay strategies to achieve multiplexed analysis of biomolecules. The combination of AuNPs with novel signal amplification methods should be further investigated to enhance the sensitivity of non-optical bioassay. To develop the practicable biosensors based on AuNPs, the operation convenience, detection time, and analysis cost have to be considered. It is possible that the non-optical biosensors with good performance should be successfully applied in the field of biomedicine.

## Figures and Tables

**Table 1:** Electrochemical biosensors based on AuNPs.

Analytes ^a^	Electrode Modification ^b^	Functions of AuNPs	Detection Limits	Ref.
*Mtb* DNA	SPCE/SA	Electrochemical indicators	1 CFU	[71]
EGFR	GCE	Electrochemical indicators	50 pg/mL	[72]
hIgG, hPSA	GCE/MWCNT/AB	Electrochemical indicators	0.3 fg/mL, 0.1 fg/mL	[73]
hMMP9	SPCE/AB	Electrochemical indicators	0.06 ng/mL	[74]
PSA	GCE/AuNPs/AB	Electron migration enhancers	145.69 fg/mL	[81]
M.SssI MTase	GCE/AuNPs/CP	Electron migration enhancers	0.04 U/mL	[82]
ErbB_2_	GE/pSC_4_/HMD/AuNPs	Electron migration enhancers	0.5 ng/mL	[84]
ssDNA	GCE/CS-GS/PANI/AuNPs/CP	Electron migration enhancers	2.11 pM	[85]
CEA	GCE/NB-ERGO/AuNPs/AB	Electron migration enhancers	1 pg/mL	[86]
MicroRNA	GCE/WO_3_-Gr/AuNPs/CP	Electron migration enhancers	0.05 fM	[87]
PDGF, TB	GCE/SWCNTs@AuNPs/AB	Electron migration enhancers	8 pM, 11 pM	[88]
PSA	PGE/GMCs@AuNPs/AB	Electron migration enhancers	0.25 ng/mL	[89]
CEA	GE/Cys/AuNPs@PAMAM/Th/AB	Electron migration enhancers	4.4 pg/mL	[90]
ssDNA	GE/CP	Immobilization platform	50 fM	[105]
Ampicillin	GCE/AuNPs/Aptamer	Immobilization platform	0.3 pM	[108]
TB	GCE/AuNPs/Aptamer	Immobilization platform	23 fM	[109]
MiRNA	GE/CP	Immobilization platform	6.8 aM	[111]
MiRNA-141	GE/CP	Immobilization platform	25.1 aM	[112]
MiRNA-182, MiRNA-381	MGE	Immobilization platform	0.2 fM, 0.12 fM	[113]
AFP	GCE/AuNPs/AB	Catalyst	3.33 fg/mL	[122]
Lysozyme	GE/CP	Catalyst	0.32 pM	[123]
Microcystin-LR	GCE/CNT/PEG	Catalyst	0.1 ng/L	[133]
PSA	GCE/Au@N-GQDs/AB	Catalyst	0.003 pg/mL	[134]
PSA	SPCE/CNT/AB	Catalyst	1.2 pg/mL	[135]

^a^*Mtb* DNA: *Mycobacterium tuberculosis* DNA; EGFR: epidermal growth factor receptor; hIgG: human immunoglobulin G; hPSA: human prostate-specific antigen; hMMP9: human matrix metallopeptidase-9; PSA: prostate-specific antigen; M.SssI MTase: methyltransferase; ErbB_2_: human epidermal growth factor receptor 2; CEA: carcinoembryonic antigen; PDGF: platelet-derived growth factor; TB: thrombin; AFP: alpha fetoprotein. ^b^ SPCE: screen printed carbon electrode; SA: streptavidin; GCE: glassy carbon electrode; MWCNT: multiwalled carbon nanotube; AB: antibody; GE: gold electrode; pSC_4_: *para*-Sulfonatocalix[4]arene; HMD: 1,6-hexanediamine; CS-GS: chitosan-graphene sheets; PANI: polyaniline; CP: capture probe; NB-ERGO: Nile blue A (NB) hybridized electrochemically reduced graphene oxide; WO_3_: tungsten oxide; SWCNTs: single-walled carbon nanotubes; PGE: pyrolytic graphite electrode; GMCs: graphitized mesoporous carbon nanoparticles; Cys: cysteamine; PAMAM: poly(amidoamine) dendrimer; Th: thionine; MGE: magnetic gold electrode; CNT: Carbon nanotubes; PEG: polyethylene glycol; Au@N-GQDs: AuNPs functionalized nitrogen-doped graphene quantum dots.
